# Fatal varicella pneumonia in an unvaccinated child with Down Syndrome: a case report

**DOI:** 10.1186/s13052-016-0312-1

**Published:** 2016-11-17

**Authors:** Diletta Valentini, Simona Bianchi, Chiara Di Camillo, Anna Chiara Vittucci, Michaela Veronika Gonfiantini, Rita De Vito, Alberto Villani

**Affiliations:** 1 Pediatric and Infectious Disease Unit, Bambino Gesù Children’s Hospital, IRCCS, Rome, Italy; 2 Pathology Unit, Bambino Gesù Children’s Hospital, IRCCS, Rome, Italy

**Keywords:** Down Syndrome, Immunodeficiency, Varicella, Pneumonia, Vaccination, Case report

## Abstract

**Background:**

Varicella is an acute infectious disease common during childhood. It has mostly an uncomplicated course in early childhood. Neverthless, it may result in severe complications, especially in particular age groups and clinical conditions. Down Syndrome represents a risk factor for developing complications, because of the frequent comorbidities and their immunodeficiency.

**Case presentation:**

A 2-year-old white Caucasian female affected by Down Syndrome was referred to our hospital for cardiac arrest in course of varicella disease. After cardiopulmonary resuscitation and stabilization, her clinical conditions didn’t improve and she developed a massive pulmonary hemorrage, which led her to exitus.

**Conclusions:**

Mortality due to varicella infection is rare, but it is more common in subjects with immune deficit or chronic pathologies, and in particular age-groups. The importance of the vaccine for preventable infectious diseases is stressed in this paper, in which we present a case of death in an unvaccinated cardiopathic child with Down Syndrome affected by varicella.

## Background

Varicella is an acute, exanthematous, highly infectious disease, that most commonly occurs in childhood. Varicella normally has a benign course, but can occasionally develop into a more serious illness, especially in adults, immunodeficient children, pregnant women, newborn babies [1]. A lethal outcome is very rare, with a mortality rate fluctuating between 0.29 and 0.46 deaths per 1 million. The introduction of the vaccine in 1995 has substantially decreased varicella incidence, hospitalizations, and deaths [2].

The most common complications of varicella are bacterial skin infection, sepsis, pneumonia, and central nervous system events such as cerebellar ataxia and encephalitis [3, 4].

## Case presentation

A 2-years-old white Caucasian female affected by Down Syndrome (DS) and surgically corrected at 3 months of age for a subaortic intraventricular defect (IVD), with a history of 2 episodes of pneumonia, was admitted in the emergency room of our hospital due to a cardiac arrest during her varicella illness. She had never been vaccinated up to the moment, that’s why she contracted the disease from her sister. The night before her admission to the hospital, she began to manifest episodes of hypotonia associated to periods of crying. At 4 o’clock she began to show signs of a generalized hypotonia and she was taken to our hospital by her parents, where she arrived in cardiac arrest. After Cardio-Pulmonary Resuscitation (CPR-PALS) her spontaneous breathing was restored. The clinical course was characterized by complete areflexia, with bilateral mydriasis. Breathing pattern was characterized by ARDS that required high frequency mechanical ventilation and Nitric Oxide with transient improvement.

**Fig. 1:**
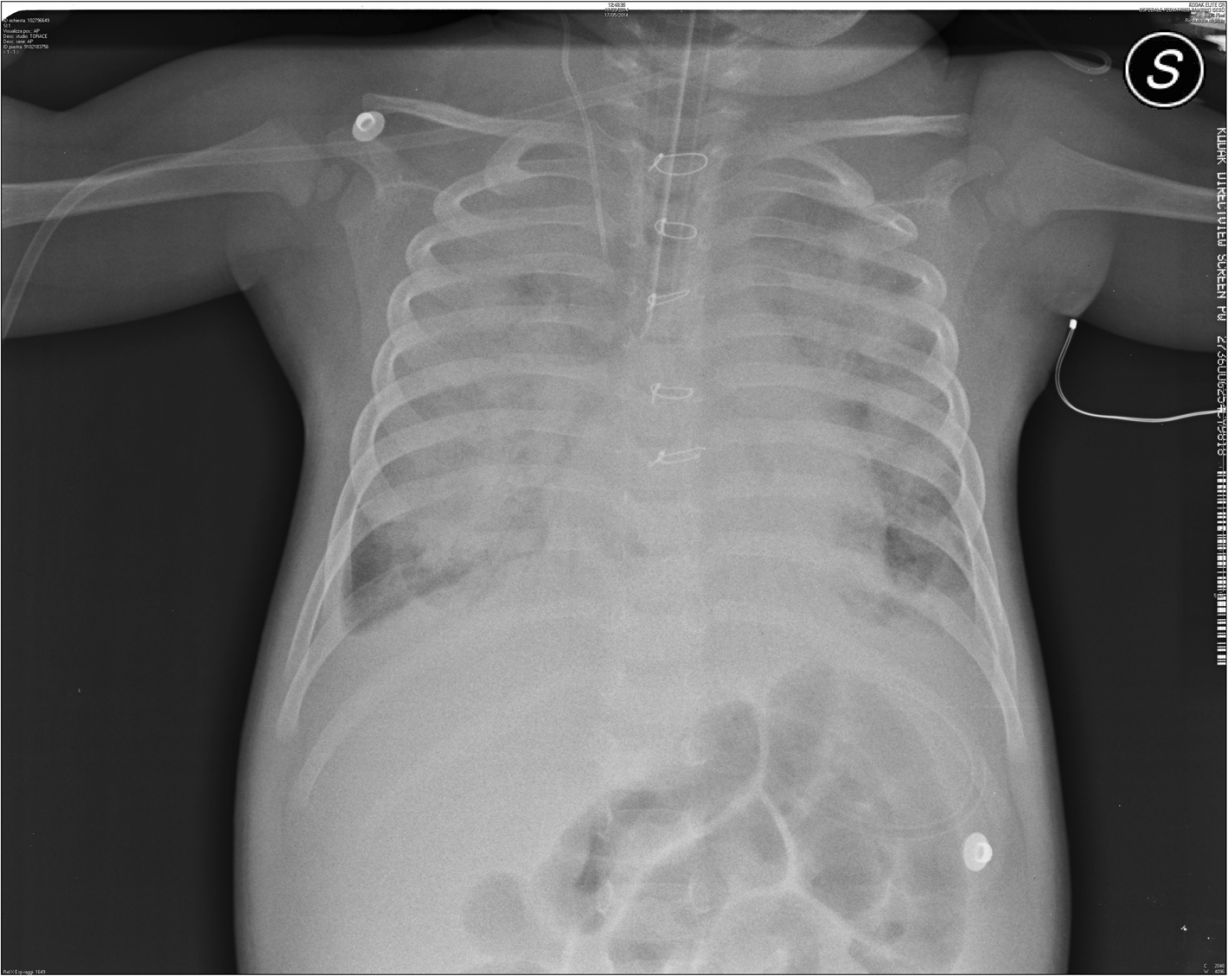
Chest X-Ray: multiple foci of parenchymal spread to both lungs and pleural effusion obliterating share of the breast-phrenic cost

We proceeded to perform a chest X-ray (CXR), which revealed multiple foci of parenchymal spread to both lungs and pleural effusion obliterating share of the breast-phrenic cost (Fig. 1). An abdominal ultrasound showed the presence of abdominal effusion in all quadrants and laboratory tests revealed the presence of IgM antibodies against varicella, positive PCR for varicella antigen, absence of bacterial infections (coltures of blood and urine), prolonged PT and PTT, and altered D-Dimer. Tests for immunological functions were performed (Table 1).

**Table 1:** Blood investigations performed at the emergency room access

Investigation	Value	Reference value
White bllod cells	15.65 (*103/uL)	5.5–15
Red blood cells	4.03 (*106/*uL)	3.6–5
Hemoglobin	9 (g/dL)	10.5–15.5
Platelets	131 (103/uL)	150–450
PTT-s	50.5 (seconds)	25–34
PTT-r	1.73 (seconds)	0.85–1.15
Trombin Time	27.6 (seconds)	16–22
Antitrombine III	34 (%)	75–120
Fibrinogen Dimeri	6.7 (microg/mL)	<0.5
LDH	4150 (UI/L)	230–470
CPK	3595 (UI/L)	32–211
CD3-pan T	56.4 (%)	58–75
CD4 T Helper	16.8 (%)	29–47
CD8 T Suppressor/Cytotoxic	39.7 (%)	17–33
CD19 Pan B	36 (%)	14–30
CD16 + CD56+	6.7 (%)	4–17
VZV PCR	positive	Negative
VZV IgG	negative	--
VZV IgM	positive	--

The next day, the respiratory condition didn’t improve and a new CXR showed an impairment of the spread, and a massive pulmonary hemorrhage. In the absence of recovery of the main indicators of organ perfusion, she was declared dead.

At macroscopic examination the lungs were heavy, firm and plum-colored, with diffuse areas of hemorrhage and necrosis.

**Fig. 2:**
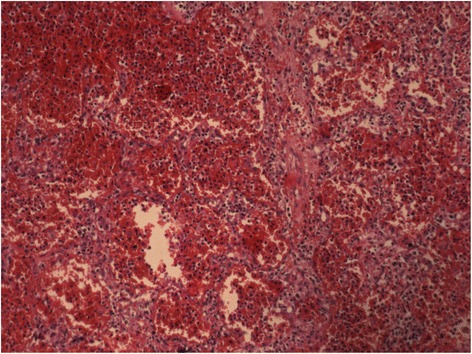
Histological image that showed interstitial pneumonitis and diffuse necrosis and hemorrhage in the pulmonary parenchyma

Histologically there are interstitial pneumonitis, diffuse necrosis and hemorrhage in the pulmonary parenchyma (Fig. 2).

## Conclusions

The estimated global burden of disease-specific mortality caused by varicella is considerably lower than that due to other major infectious diseases such as measles, pertussis, rotavirus, or invasive pneumococcal disease. Based on conservative estimates, the global annual varicella disease burden reports 4.2 million severe complications leading to hospitalization and 4200 deaths [5].

The prevalence of immunocompromising conditions including HIV infection and the kind of treatment available, are factors which influence the course of the disease. In healthy children, varicella is usually self-limiting and benign [6].

Groups at higher risk for severe complications are: neonates, infants, pregnant women, adults, and immunocompromised patients.

The type of varicella complications depends on the patient’s age. A study of Gowin et al. demonstrates that the average age of varicella-complications in hospitalized children is 3.12 years [7, 8]. The youngest children had pneumonia, and the oldest meningitis/meningoencephalitis [9, 10].

Older children considered to be more susceptible to antibody-mediated inflammatory reactions, whereas younger, less immunocompetent patients are more frequently affected by bacterial suprainfections of the skin or of the respiratory tract [11, 12].

Respiratory tract infections were present in younger children, and usually developed at the beginning of the varicella infection [7]. The high frequency of respiratory tract complications reflects the biology of the virus. The virus enters the host through the respiratory tract and then spreads in the bloodstream. A cytopathic effect of the varicella virus on the alveolar epithelium causes pneumonia. Desquamated alveolar cells contribute to reduce gas exchange.

Hematological complications are observed in 1–2% of children with varicella. Patients usually remain asymptomatic. Like many other thrombocytopenias and anemias during viral infections, those in patients with varicella are transient and require no treatment. Laboratory tests are not performed routinely in patients with varicella [13].

Our case suffered from acute respiratory distress syndrome (ARDS) caused by varicella, and associated to hematological disorders that provoked the development of pulmonary hemorrhage, which caused death.

Children who are diagnosed with Down syndrome and who have comorbidities such as immunodeficiency or cardiopathy have high rates of viral and bacterial infections such as influenza and pneumococcal infections [14]. The most common symptoms reported in children with DS are infections of the respiratory tract suggesting a B-cell defect [15]. Diseases related to T-cell deficiency, such as infection with intra-cellular microorganism, fungi and opportunistic pathogens are rare [16].

Varicella is one of the most infective disease that affects the pediatric population.

Guidelines stress the importance of an anti-varicella vaccination for all children older than 12 months as well as children with DS [17]. The only contraindication of the vaccine is severe immunodeficiency of the T cells [6]. Despite the fact that most children hospitalized with varicella complications were immunologically healthy, risks are superior in subjects with chronic conditions [6, 18].

Children with DS have a higher risk of being hospitalized for viral respiractory tract infections, even in the absence of coexisting risk factors [19], and the mortality rate in severe ill DS children admitted for medical reasons is high and is predominantly associated with respiratory conditions [20]. As the burden of preventable infections in children diagnosed with chronic diseases is high in terms of incidence and severity, it is essential to protect these children with timely administration of vaccinations according to the current recommendations.

Even among populations at risk, varicella vaccine is the less used. Although Italian Down children have the best vaccination coverage among all patients with chronic disease [21], it is clear that it is desirable to improve vaccination coverage among risk groups and also among the healthy population.

Implemented interventions, with special reminders to parents and general practitioners, are necessary to promote timely vaccination in DS children who are susceptible to preventable infections and prone to severe complications.

This case underlines how varicella may lead to severe, potentially life-threatening complications in unvaccinated children and adolescents, and may demonstrate too the benefits of varicella vaccination.

## Abbreviations

ARDS: Acute respiratory distress syndrome
CPR-PALS: Cardio-Pulmonary Resuscitation
CXR: Chest X-ray
DS: Down Syndrome
